# Effects of five years conservation tillage for hedging against drought, stabilizing maize yield, and improving soil environment in the drylands of northern China

**DOI:** 10.1371/journal.pone.0282359

**Published:** 2023-03-06

**Authors:** Zizheng Deng, Mingjing Huang, Wuping Zhang, Guofang Wang, Xuefang Huang, Gaimei Liang, Nana Li

**Affiliations:** 1 College of Resources and Environment, Shanxi Agricultural University, Taigu, Jingzhong, Shanxi, China; 2 Shanxi Institute of Organic Dryland Farming, Shanxi Agricultural University, Taiyuan, Shanxi, China; 3 State Key Laboratory of Integrative Sustainable Dryland Agriculture (in preparation), Shanxi Agricultural University, Taiyuan, Shanxi, China; 4 College of Software, Shanxi Agricultural University, Taigu, Jingzhong, Shanxi, China; University of Minnesota, UNITED STATES

## Abstract

Continuous tillage cultivation positioning trials can provide the basis for maintaining soil health, improving resource utilization efficiency and crop productivity, and achieving sustainable agricultural development. In this study, changes in soil stability and water–holding capacity characteristics were measured under different tillage cultivations from a multi–year microscopic perspective and analyzed to evaluate selected key indicators. Continuous monitoring of rainfall utilization efficiency and yield was carried out for five years. Here, we discuss the role of conservation tillage in buffering and stabilizing rainfall precipitation pattern on the fluctuation and uncertainty of soil water retention and water supply capacity and soil quality. The study was carried out on dryland areas of the Loess Plateau in northern China with eight tillage systems established in 2016: no–tillage (NT); no–tillage and straw (NTS); subsoiling (SU); subsoiling and straw (SUS); rotary tillage (RT); rotary tillage and straw (RTS); conventional tillage (CT); and conventional tillage and straw (CTS). All treatments were applied in conjunction with continuous cropping for five years. The evaluated soil parameters were mean weight diameter (MWD), geometric mean diameter (GMD), >0.25 mm aggregate content (R_0.25_) of water–stable aggregates (WSAs), soil moisture characteristic curve (SMCC), specific soil water capacity (*C*_*θ*_), soil organic matter (SOM), rainfall utilization efficiency (RUE), and maize yields for five consecutive years. The MWD, GMD, and R_0.25_ of SUS were 27.38%, 17.57%, and 7.68% more than CTS (control), respectively. Overall, SOM, average annual RUE, and average annual yields increased by 14.64%, 11.89%, and 9.59%, respectively, compared with 2016. Our results strongly suggest that conservation tillage can considerably improve these characterization indicators. SUS was more effective than CTS in the 0–40 cm soil layer at hedging against drought in the area, stabilizing crop production, and achieving sustainable agricultural development.

## 1. Introduction

Global arid areas (or drylands) account for approximately 45% of the earth’s land surface [1]. Since a large area of China is in the mid–latitude zone, arid areas account for approximately 70% of the country’s land area, of which arid and semi–arid arable land area accounts for 43% of the total arable land area [2]. Furthermore, the climate and soil conditions in most part of China are suit able for growing maize, making China the second largest maize producer in the word, what’s more, on the arid arable land, the area planted with maize accounts for approximately two–thirds of the total sown area [3]. Declining production of maize, a major global food crop, could threaten global food security [4]. However, higher levels of organic matter in soil can improve soil water retention and water use efficiency and mitigate agricultural yield losses caused by drought [5]. Soil aggregates are soil structures with various excellent functions, and as such, they play a crucial role in SOM, which solves imbalances between water, fertilizer, gas, and heat in the soil through optimization of the soil three–phase ratio; it further indirectly improves crop productivity through its impact on drought resistance, moisture conservation, and reduction of surface runoff [6].

Soil structure and agricultural conditions that affect crop production are related to the manner and degree of land use by humans. The use of conventional tillage (CT) in cultivation disrupts soil structure and reduces crop productivity. In contrast, using conservation tillage in cultivation often enhances soil structural stability and increases crop productivity [7]. Therefore, conservation tillage cultivation systems have the potential to perfect soils by improving aggregate structure, enhancing soil moisture storage, retention, and supply ability, and providing efficient SOM functions compared to CT [8]. The adoption of conservation tillage cultivation systems has increased worldwide due to its excellent performance in terms of soil health, agricultural resource utilization, crop productivity, and sustainable agriculture.

Conservation tillage refers to a sustainable agricultural technology that can reduce soil erosion, improve precipitation utilization, and encourage the coordinated development of ecological, economic, and social benefits [9]. At present, conservation tillage cultivation with a high penetration rate mainly includes no–tillage (NT), subsoiling (SU), rotary tillage (RT), and various forms of tillage with straw returning.

Soil structure is linked with several agronomic and environmental processes [10]. Many studies of conservation agricultural management systems have found that NT resulted in a lower degree of compactness in the 0–0.15 cm (86%) and 0.15–0.30 cm (78%) soil layers and a higher maize yield than CT [11]. Moreover, NT cultivation can improve soil aggregate formation and stability, porosity, and organic carbon levels [12]. SU increased the amount of available water in the soil by reducing surface runoff, promoting crop growth, yield, and rainfall utilization efficiency (RUE), and improving the soil structure and physical properties. Furthermore, SU can be used on thick and compacted soil to reduce further soil compactness and increase plant productivity [13,14]. RT can effectively improve soil structure, reduce soil bulk density, and increase crop productivity [5,15]. Straw return can significantly reduce soil bulk density and increase SOC content, storage capacity, and the proportion of large aggregates (R_0.25_), as well as their stability in the bottom plow layer [16].

To understand the benefits of conservation cultivation converted from conventional cultivation of soil, many experts in the field of agriculture have conducted research from multiple perspectives [17,18]. However, there is comparatively less information available on the simultaneous effects of eight tillage practices on soil structure, fertility, water supply, water retention, and crop productivity. This present study was conducted based on the hypothesis that in the arid regions of northern China, conservation cultivation can improve soil physical and hydrological properties. Our objectives were to (1) assess the impacts of no–tillage (NT), no–tillage and straw (NTS), subsoiling (SU), subsoiling and straw (SUS), rotary tillage (RT), rotary tillage and straw (RTS), conventional tillage (CT), and conventional tillage and straw (CTS) on water–stable aggregates (WSAs), soil water holding and supply capacity, soil organic matter (SOM), and rainfall use efficiency (RUE) and (2) investigate the effectiveness of tillage practices on rainfall utilization efficiency and food security under different rainfall precipitation pattern.

## 2. Materials and methods

### 2.1 Study site and treatments

This study was conducted as part of a long–term experiment in the Jinzhong National Long–term Test Observation Platform in Loess Plateau, China (37°32′44.28″N, 112°37′26.78″E). The test area had a temperate continental monsoon climate with an average annual temperature of 9.8°C. The average annual rainfall is approximately 400–450 mm, and more than 70% of the rainfall occurs toward the end of the second quarter in June and during the entire third quarter of the year. The average annual frost–free period is 158 days, and ≥10°C, the annual accumulated temperature is approximately 3600°C. The soil type is soil with a pH of 8.0, and the basic soil physical properties are summarized in Table 1. The amounts of organic matter, total nitrogen, available nitrogen, available phosphorus, and available potassium were 17.4 g kg^–1^, 1.95 g kg^–1^, 119.5 mg kg^–1^, 11.6 mg kg^–1^, and 241.9 mg kg^–1^, respectively.

**Table 1:** Basic physical soil properties of the experimental site.

Soil depth (cm)	Soil texture	Bulk density (g cm^3^)	Field capacity (%)	Wilting point (%)
0–20	Clay soil	1.22	32.7	11.6
20–40	Clay soil	1.47	30.9	14.0

**Fig 1:**
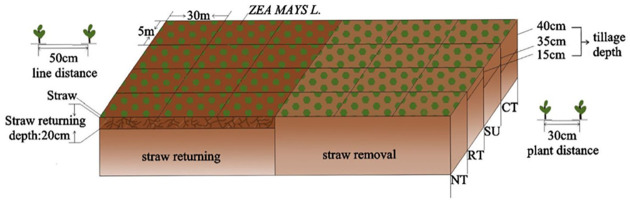
Detailed description of different tillage methods.

The field experiment began in 2016 and consisted of eight treatments on plots with areas of 150 m^2^ (5 m × 30 m) per plot. Each treatment was applied to three plots, resulting in a total of 24 plots. The eight treatments were NT, NTS, SU, SUS, RT, RTS, CT, and CTS (Fig 1). Detailed information about the treatments and sites is presented in Table 2. A continuous cropping system was adopted for planting once annually for five years using the test crop spring maize (Dafeng "30"). The planting density was 60,000 plants ha^–1^. The land was cultivated annually in late April using different methods, trial plots were weeded from the seedling to the jointing stage, and combined harvesting was performed in late October.

**Table 2:** Details of tillage treatments.

Treatment	Description	Crop straw handling
NT	No mechanical disturbance to the soil except for seeding with a maize planter and harvesting with a harvester.	After the maize has been harvested, all crop residues are removed.
SU	In addition to the seeder and harvester, the soil is loosened by machinery before maize planting in the 0–30 cm soil layer without changing the soil layer sequence.	After the maize has been harvested, all crop residues are removed.
RT	In addition to the seeder and harvester, the soil is loosened by mechanical rotary breaking of the 0–15 cm soil layer before planting.	After the maize has been harvested, all crop residues are removed.
CT	In addition to the seeder and harvester, the 0–40 cm soil layer is mechanically shoveled, broken up, and thinned, and the soil layer sequence is changed.	After the maize has been harvested, all crop residues are removed.
NTS	No mechanical disturbance to the soil except seeding with a corn planter and harvesting with a harvester.	After the maize has been harvested, all straw residues are crushed to approximately 10 cm long and covered on the ground during recreation.
SUS	In addition to the seeder and harvester, maize is loosened by machinery before planting in the 0–30 cm soil layer, without changing the soil layer sequence.	After the maize has been harvested, all straw residues are crushed to approximately 10 cm long and mulched on the surface during recreation and the following year with deep loosening of the 0–30 cm soil layer mixed into the soil.
RTS	In addition to the seeder and harvester, the soil is loosened by mechanical rotary breaking of the 0–15 cm soil layer before planting.	After the maize has been harvested, all straw residues are crushed to approximately 10 cm long and covered on the surface during recreation, and the following year with the rototilling of the 0–15 cm soil layer mixed into the soil.
CTS	In addition to the seeder and harvester, the 0–40 cm soil is mechanically shoveled, broken up, and thinned and the soil layer sequence is changed.	After the maize has been harvested, all straw residue is crushed to approximately 10 cm long and covered on the surface during recreation, and the following year with the ploughing tillage of the 0–40 cm soil layer mixed into the soil.

Note: NT, no–tillage; SU, subsoiling; RT, rotary tillage; CT, conventional tillage; NTS, no–tillage and straw; SUS, subsoiling and straw; SUS, subsoiling and straw; RTS, rotary tillage and straw; CTS, conventional tillage and straw.

### 2.2 Sample collection

Soil change is a slow process; it takes approximately 4–5 years to effectively convert traditional tillage into conservation tillage [19]. On September 28, 2020, we randomly collected mixed soil samples from five points of each plot. Undisturbed soil samples of the 0–10 cm, 10–20 cm, 20–30 cm, and 30–40 cm soil layers were collected by drilling soil with stainless steel rings (100 cm^3^ volume) for soil aggregate and soil moisture characteristic curve analyses. Rainfall data from the study area were obtained from weather stations, and the weighted Markov chain [20] was used to determine the annual rainfall precipitation pattern. The grading standards are shown in Table 3 and the rainfall precipitation patterns from 2016 to 2020 are shown in Table 4.

**Table 3:** Rainfall precipitation pattern classification table.

Serial number	Rainfall precipitation pattern	Grading standards
1	Low rainfall year	** $\text{X}<\bar{\text{X}}-1.1*\text{S}$ **
2	lower than average rainfall year	** $\bar{\text{X}}–1.1*\text{S}\leq \text{X}<\bar{\text{X}}–0.5*\text{S}$ **
3	Normal rainfall year	** $\bar{\text{X}}-0.5*\text{S}\leq \text{X}<\bar{\text{X}}+0.5*\text{S}$ **
4	Higher than average rainfall year	** $\bar{\text{X}}+0.5*\text{S}\leq \text{X}<\bar{\text{X}}+1.1*\text{S}$ **
5	High rainfall year	** $\text{X}\geq \bar{\text{X}}+1.1*\text{S}$ **

Note: $\bar{\text{X}}$, 2016–2020 average annual rainfall precipitation; S, standard deviation; X, rainfall precipitation in a year.

**Table 4:** Annual rainfall precipitation pattern from 2016 to 2020.

Year	2016	2017	2018	2019	2020
**Rainfall precipitation pattern**	Higher than average rainfall year	Normal rainfall year	Lower than average rainfall year	Low rainfall year	Higher than average rainfall year

### 2.3 Characterization index determination

The aggregate size distribution of the soil was measured using a wet sieving method. For the aggregate analysis, five sieves were placed into the DIK–2012 constant temperature soil aggregate analyzer apparatus (Shanghai Zequan Technology Co., Ltd. Shanghai, China). According to the instrument manual, 50 g of air–dried soil sample was used for wet sieving in deionized water. The soil aggregates in sieves are moisturized slowly by stroking; the length of each stroke was 38 mm, and the frequency was 30 strokes/min. After 30 min, five aggregate size fractions were collected (>2 mm, 1–2 mm, 0.5–1 mm, 0.5–0.25 mm, and 0.25–0.106 mm). The soil aggregates in each sieve were oven–dried at 105°C for 8 h and then weighed. The data were analyzed to calculate water–stable aggregates (WSAs), which included mean weight diameter (MWD), geometric mean diameter (GMD), and >0.25 mm aggregate content (R_0.25_). The MWD and GMD were calculated as follows [11]:

$$\text{MWD}=\overset{\text{n}}{\underset{\text{i}=1}{\sum }}\left(x_{i}m_{i}\right)$$ (1)

$$\text{GMD}=\text{EXP}\left[\frac{\sum_{\text{i}=1}^{\text{n}}\;{\text{m}}_{\text{i}}\;xlogx_{i}}{\sum_{i=1}^{n}\;m_{i}}\right]$$ (2)

where n is the number of aggregate size fractions, *x*_*i*_ is the mean diameter of the ith size class (mm), and m_i_ is the proportion of the total sample (WSA) in the corresponding size fraction.

R_0.25_ was calculated as follows [21]:

$$R_{0.25}=\frac{M_{T>0.25}}{M_{T}}$$ (3)

where R_0.25_ is the particle size >0.25 mm agglomerate weight and *M*_*T*_ is the total weight of the agglomerate.

According to the instruction manual of the Hitachi CR22N high–speed constant temperature refrigerated centrifuge (Hitachi Koki Co. Tokyo, Japan), the stainless steel rings were placed into a custom centrifuge box and eight gradient speeds were set to measure soil water suction, which was calculated as follows [22]:

$$S=\frac{\rho_{w}\omega^{2}}{2g}\left(R_{1}^{2}-R_{2}^{2}\right)$$ (4)

where S is the soil water suction (cm), *R*_1_ is the distance between the centrifuge rotor axis and the soil center (cm), *R*_2_ is the distance between the centrifuge rotor axis and the bottom of the soil sample (cm), *ρ*_*w*_ is the water density (g/cm^3^), *g* is the acceleration of gravity (cm/s^2^), and *ω* is the angular velocity (rad/s).

The structure fitting model is commonly used to identify the relationship between the matrix potential of soil moisture and water content. The model is based on different soil textures in China and has excellent effects and a wide range of applications [23]. The specific water capacity is a quantitative index of soil water release, which can effectively reflect the water supply capacity of the soil. The larger the specific water capacity, the stronger the water supply capacity [24]. The formula is as follows:

$$\theta =aS^{-b}$$ (5)

where S is the soil water suction (cm), θ is the soil volumetric water content (%), and a and b are parameters.

Cθ=−dθdS=abS−b+1 (6)

where *C*_*θ*_ is specific water capacity of soil, *θ* is soil moisture, S is soil water suction, and a and b are constants.

The measurement and calculation methods of production are consistent with Liu et al. [25]. RUE was calculated using the following equation:

$$RUE=\frac{Y}{PG}$$ (7)

where RUE is rainfall utilization efficiency (kg ha^–1^ mm), Y is the grain yield (kg ha^–1^), and PG is precipitation during the growing season (mm).

SOM was determined using the potassium dichromate volumetric method [26].

### 2.4 Statistical analysis

Data were processed using Microsoft Excel 2019. One–way analysis of variance (ANOVA) was conducted to compare the effect of different treatments. The effects of the same tillage cultivations on different straw returning methods were identified using SPSS 23.0 software. Statistical significance was determined at *P* = 0.05 [27]. The relationship between characterization indexes was determined using Pearson’s correlation matrix in RStudio [28]. Diagrams and tables were drawn using AUTOCAD 2019, Origin 2019, and Adobe Illustrator 2022.

## 3. Results

In this study, a total of eight tillage cultivations were tested. CTS was the control for the straw mulching treatment, CT was the control for the straw removal treatment, and under the same tillage with straw removal as control to study the coupling effect of straw returning and tillage.

### 3.1 Effects of tillage cultivation on soil aggregate stability

**Fig 2:**
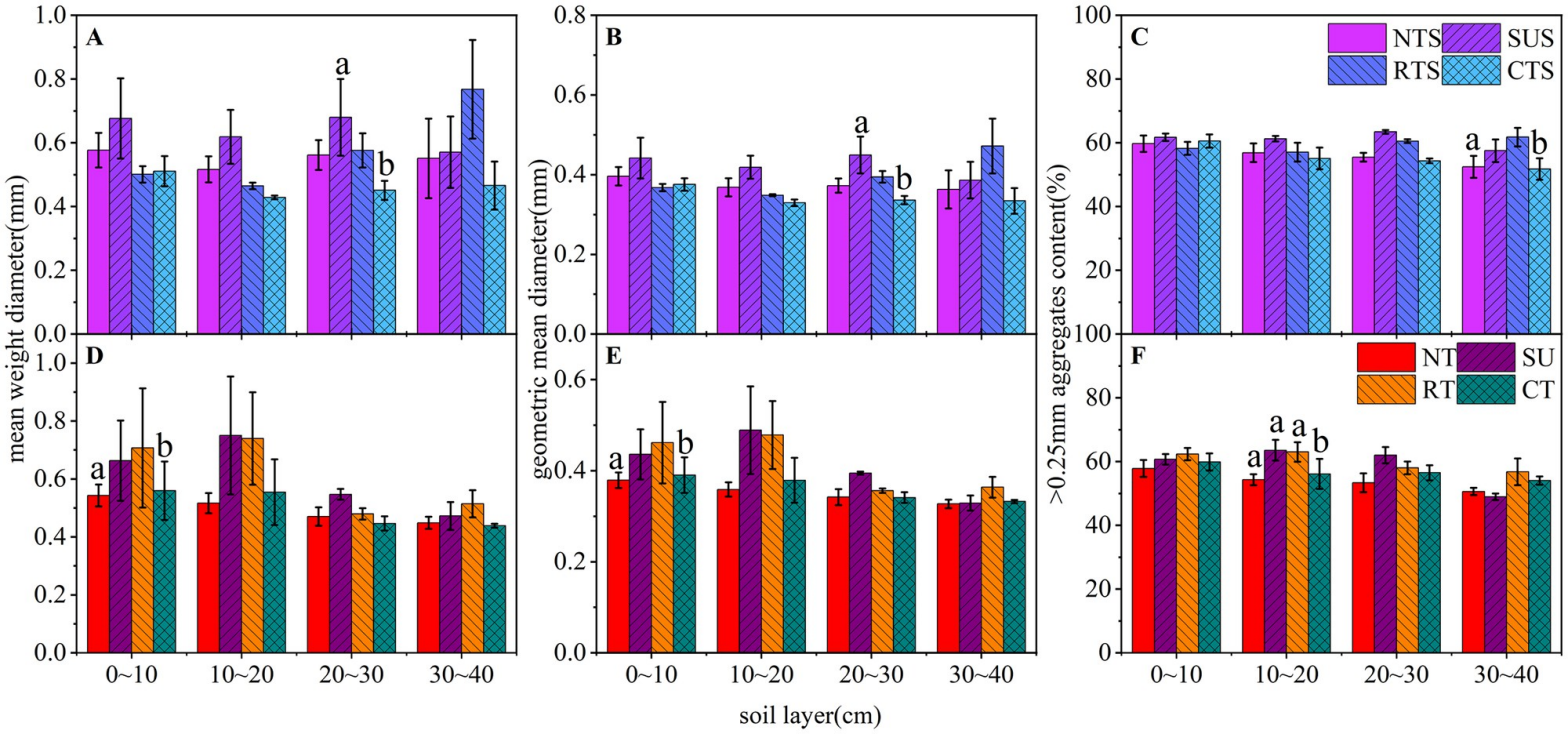
Effects of tillage cultivation on MWD, GMD, and R_0.25_ in the 0–40 cm soil layer (2020). Note: The error bars indicate a significant difference at the *P* < 0.05 level (two–tailed test).

The stability of WSAs were measured using MWD, GMD, and R_0.25_ (Fig 2), using a method consistent with Zhang et al. [29]. In the 0–40 cm soil layer, tillage significantly affected the stability of soil aggregates. In the 20–30 cm soil layer, compared with CTS, SUS significantly increased the MWD and GMD of WSAs by 50.84% and 33.75%, respectively, and NTS significantly increased R_0.25_ in the 30–40 cm soil layer by 1.35% compared to the CTS. Moreover, in the 0–30 cm soil layer under SUS, the MWD, GMD, and R_0.25_ of WSAs were the highest. Compared with CT, NT significantly reduced the MWD and GMD of WSAs in the 0–10 cm soil layer by 2.9% and 2.8%, respectively, and in the 10–20 cm soil layer, the R_0.25_ significantly decreased by 3.19%. In SU and RT, R_0.25_ significantly increased by 13.25% and 12.29% in the 10–20cm soil layer, respectively.

**Fig 3:**
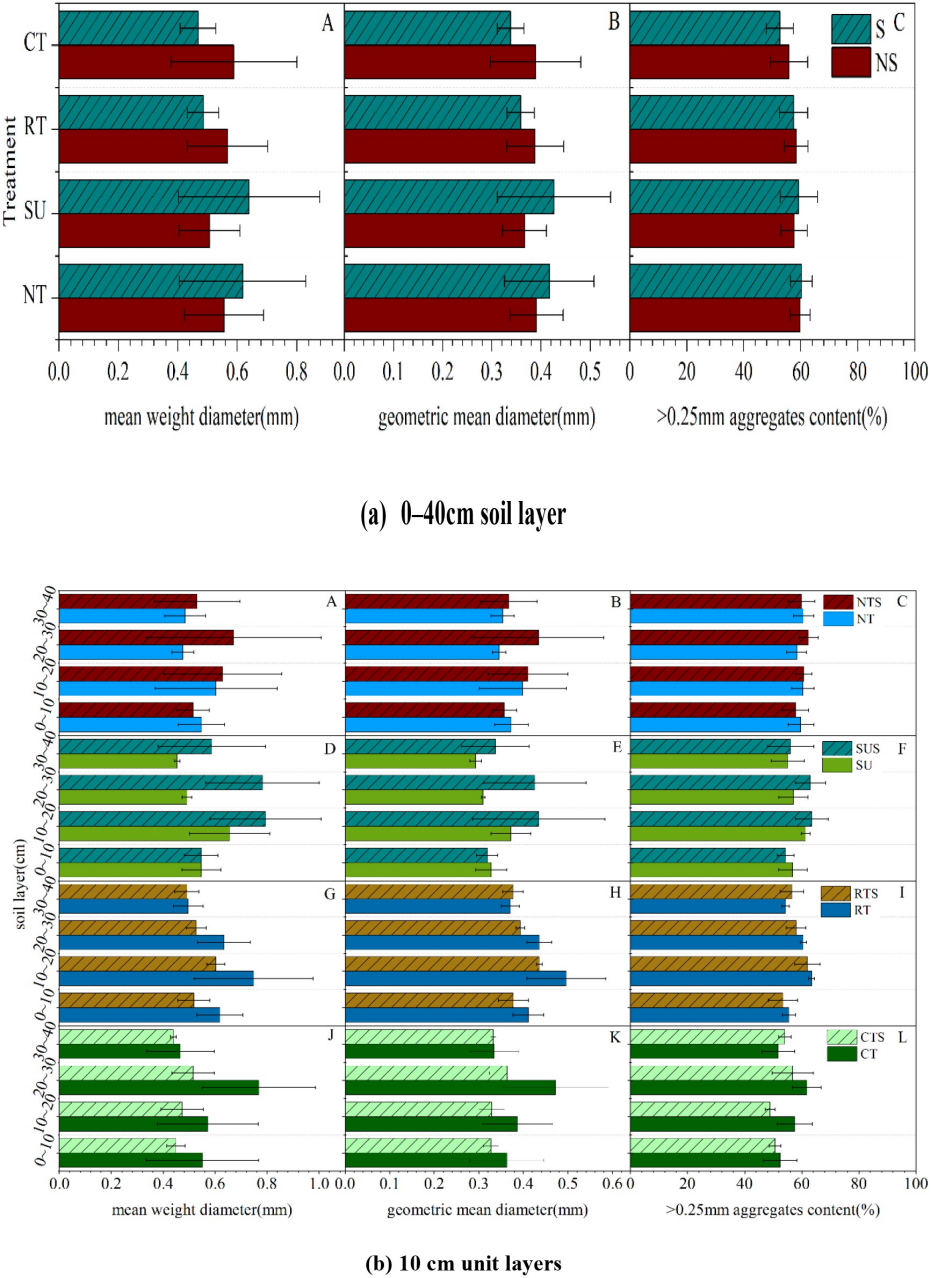
Comparison of the effects of straw return on MWD, GMD, and R_0.25_ under the same tillage cultivations. Note: S, straw return; NS, straw removal.

Under the same tillage cultivation, no significant effects of straw return were observed on the stimulation of WSAs (Fig 3(a)). A comparison of straw removal treatments in terms of the MWD, GMD, and R_0.25_ of WSAs provided the following results: The coupling effect of NT, SU, and straw returning decreased MWD, GMD, and R_0.25_ by 0.5–6.5% and 2.5–21.0%, respectively. The coupling effect of RT, CT, and straw returning increased MWD, GMD, and R_0.25_ by 2.5–17.0% and 6–26.0%, respectively.

The effects of straw returning on WSAs were analyzed in 10 cm unit layers (Fig 3(b)). Under NTS and SUS, the MWD, GMD, and R_0.25_ of WSAs increased at 0–10 cm and decreased considerably in the 10–30 cm and 30–40 cm soil layers. For NTS alone, R_0.25_ increased by 1.81% compared to NT. Under RTS and CTS, the MWD, GMD, and R_0.25_ of WSAs increased at 0–30 cm; the WSAs index obeyed a random distribution in the 30–40 cm soil layer compared to RT and CT.

### 3.2 Effects of tillage cultivation on soil water holding and supply capacity

#### 3.2.1 Soil–moisture characteristic curve

**Fig 4:**
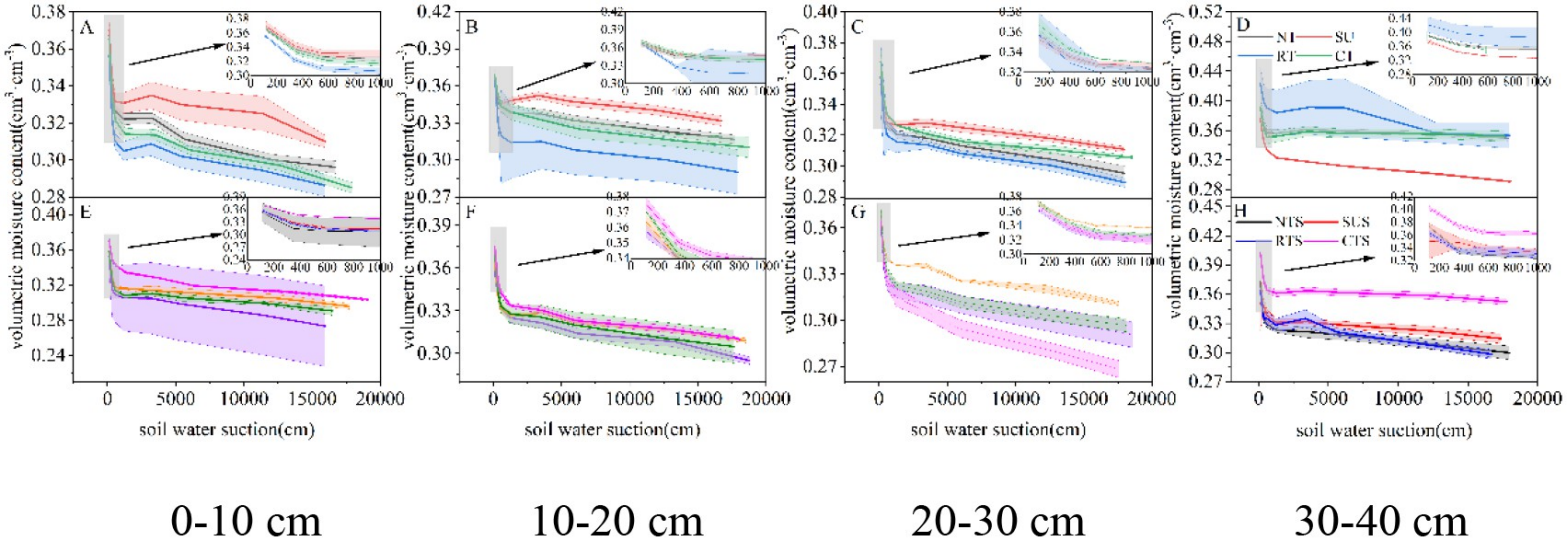
Change in SMCC in each soil layer with different tillage cultivations.

The soil moisture characteristic curve reflects the relationship between soil water and energy [23]. Under the same water suction, the higher the curve, the greater the soil volumetric water content, the stronger the water–holding capacity, and vice versa [30]. The SMCC was significantly affected by different water pressure treatments in the same soil layer (Fig 4).

With straw return under the same water suction, we observed that under CTS and SUS at 0–20 cm, SUS and RTS at 20–30 cm, and CTS and SUS in the 30–40 cm soil layer had substantial water–holding capacity. With straw removal, under the same water suction, we observed that under SU and NT at 0–20 cm, SU and CT at 20–30 cm, and RT and NT in the 30–40 cm soil layer had substantial water–holding capacity.

**Fig 5:**
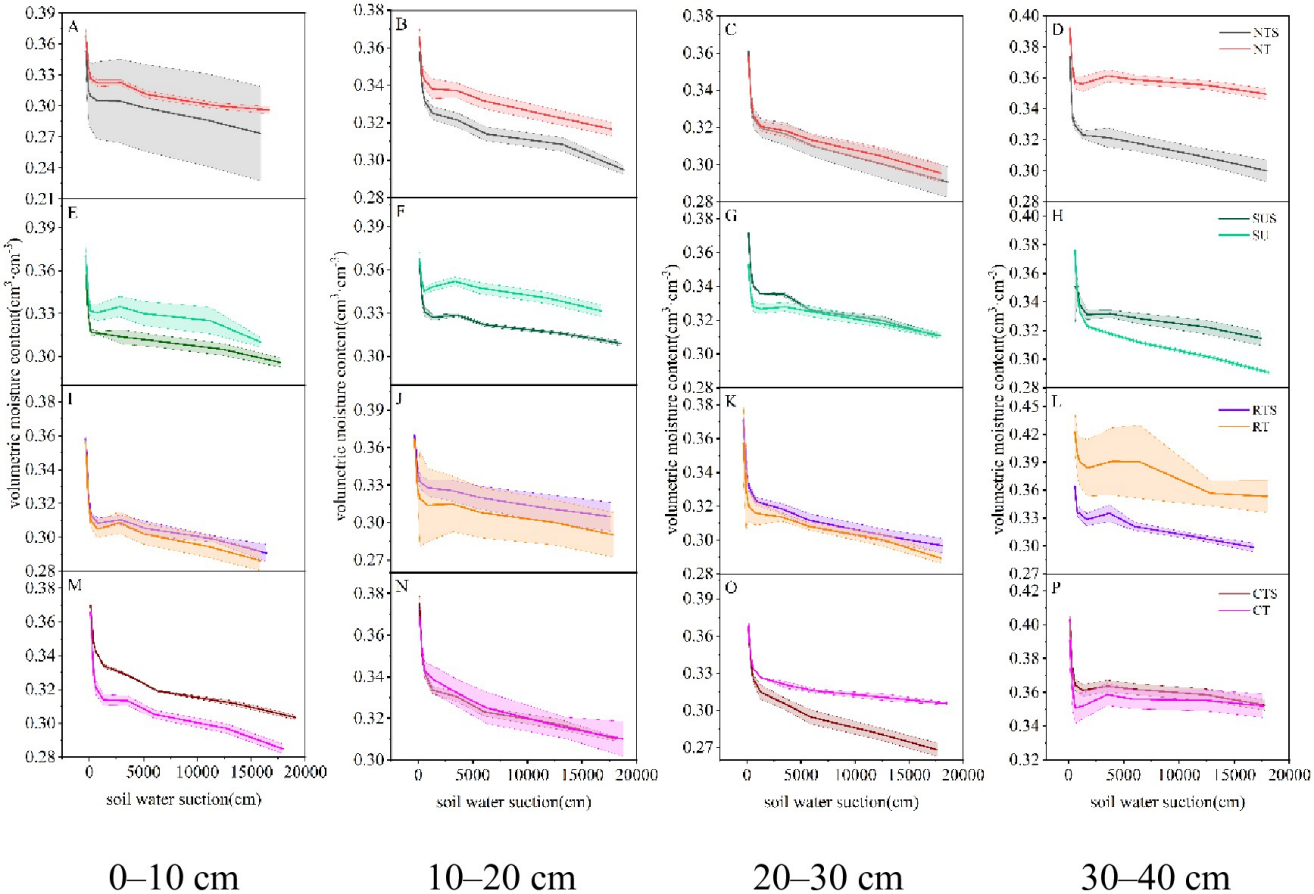
Effect of straw return on the SMCC of each tillage cultivation in each soil layer.

We analyzed the influence of straw return on each tillage cultivation on the SMCC of different soil layers (Fig 5). With the same water suction, NT at 0–40 cm, SU at 0–20 cm, RT at 30–40 cm, and CT at 10–30 cm had greater soil water–holding capacity for straw removal than straw return. Conversely, the soil water–holding capacity of straw return was greater than that of straw removal for SU at 20–40 cm, RT at 0–30 cm, and CT at 0–10 cm and 30–40 cm.

#### 3.2.2 Soil–water supply capacity

**Fig 6:**
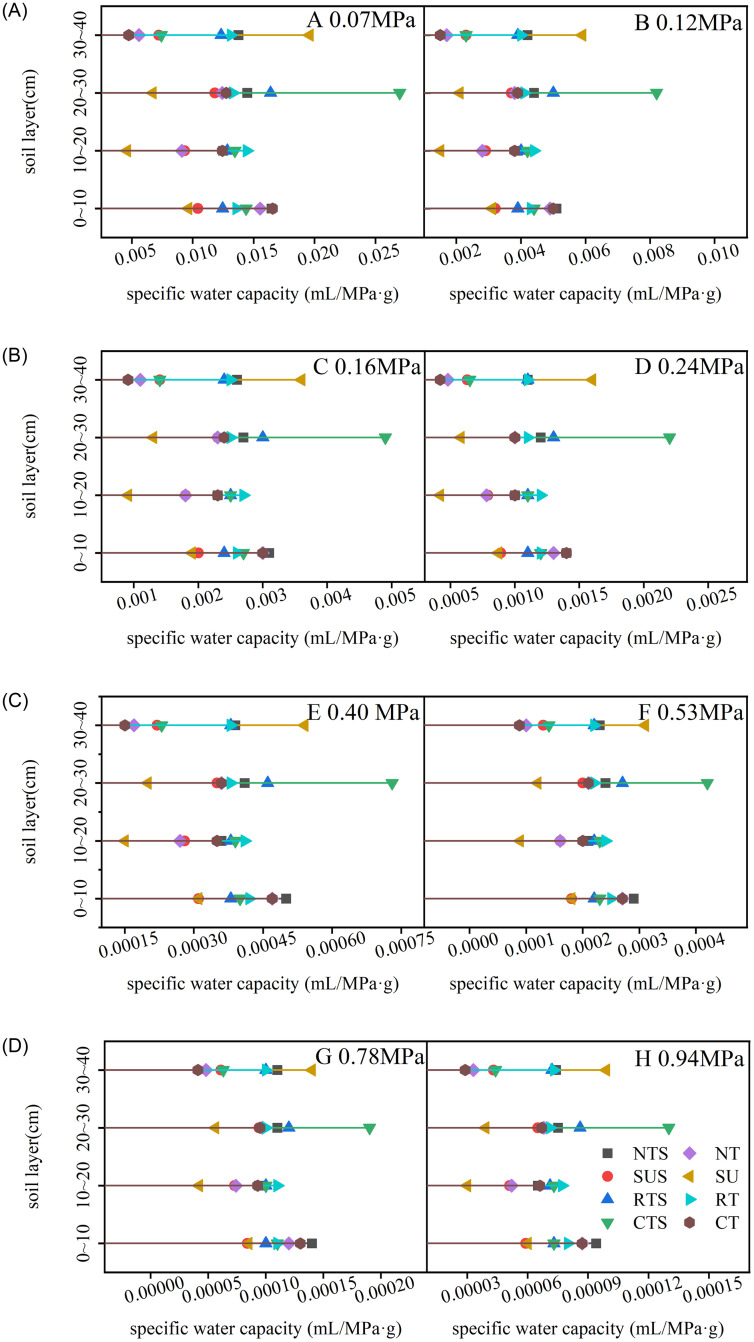
Soil specific water capacity of different soil layers under different tillage cultivations at different soil water suction levels. **Note**: At the same soil suction, the larger the specific water capacity, the stronger the water supply capacity. NT, no–tillage; SU, subsoiling; RT, rotary tillage; CT, conventional tillage; NTS, no–tillage and straw; SUS, subsoiling and straw; SUS, subsoiling and straw; RTS, rotary tillage and straw; CTS, conventional tillage and straw.

Tillage cultivation and soil suction influence soil–water supply capacity [31] (Fig 6). Under different tillage cultivations, the soil–water supply capacity continued to decline with the continuous increase of soil suction. At 0.07 MPa suction, CT had a strong water supply capacity in the 0–10 cm soil layer, while at all other suction levels, NTS soil had a strong water supply capacity. In different soil layer, RT had strong water supply capacity at 10–20 cm, CTS at 20–30 cm, and SU at 30–40 cm.

### 3.3 Effects of tillage cultivation on soil organic matter

**Fig 7:**
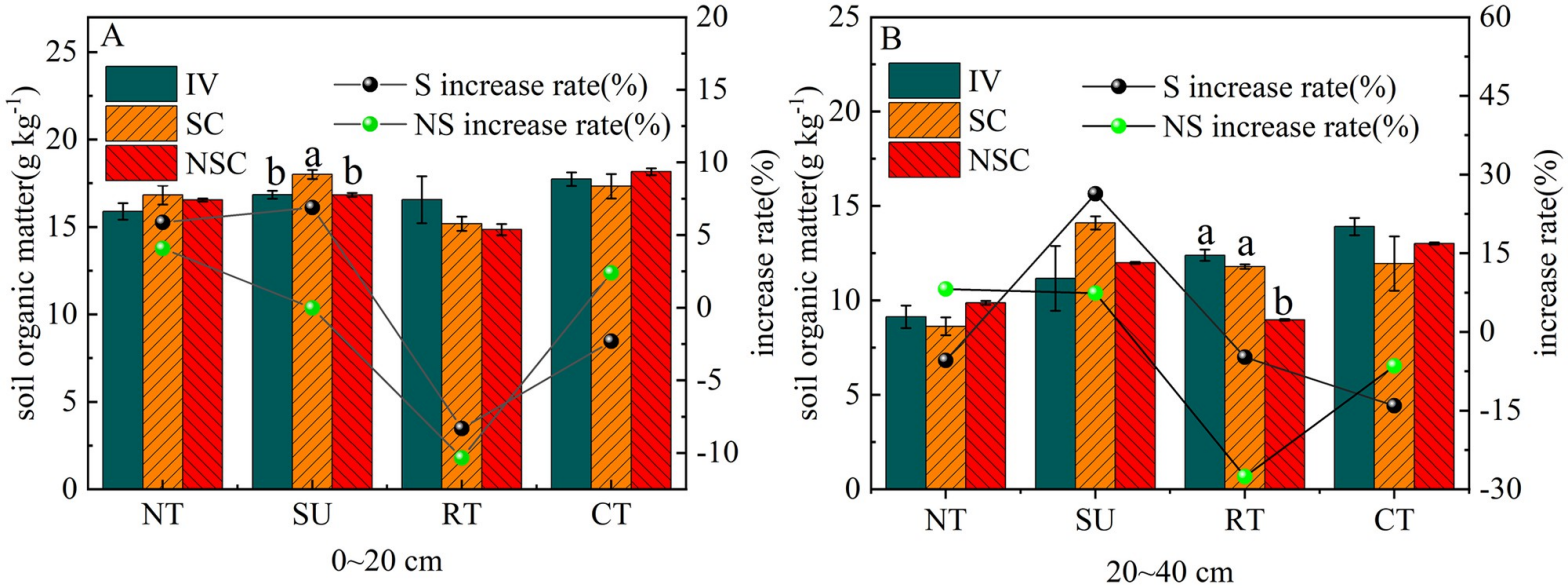
Variation of SOM in different tillage cultivations and soil layers. Note: IV, SOM content in 2016; S, SOM content in 2020 with straw return; NS, SOM content in 2020 with straw removal; SC: Straw cover return; NSC: No straw cover return. The left coordinates represent the comparison of SOM content between the starting year of the positioning experiment and after five years of a continuous cultivation. The right coordinates represent different treatments and the increase in SOM compared with the starting year of the positioning experiment.

In the arid region of northern China, 0–20 cm is regarded as the cultivation horizon, and 20–40 cm is regarded as the plow pan; therefore, changes in soil organic matter were analyzed under different tillage cultivations in 20 cm increments [29]. Compared to the beginning of the experiment (2016) (Fig 7), the 0–20 cm soil layer under NTS, SUS, NT, SU, and CT, and in the 20–40 cm soil layer under NT, SUS, and SU had improved SOM to varying extents after five years of cultivation. Among these, SUS was the most effective tillage cultivation for SOM improvement; in the 0–20 cm soil layer, SOM significantly increased by 6.90% (*P* < 0.05), while in the 20–40 cm soil layer SOM increased by 26.32%. Furthermore, under RT, SOM significantly increased by 27.60% in the 20–40 cm soil layer.

### 3.4 Effects of tillage cultivation on rue and yield characteristics

**Fig 8:**
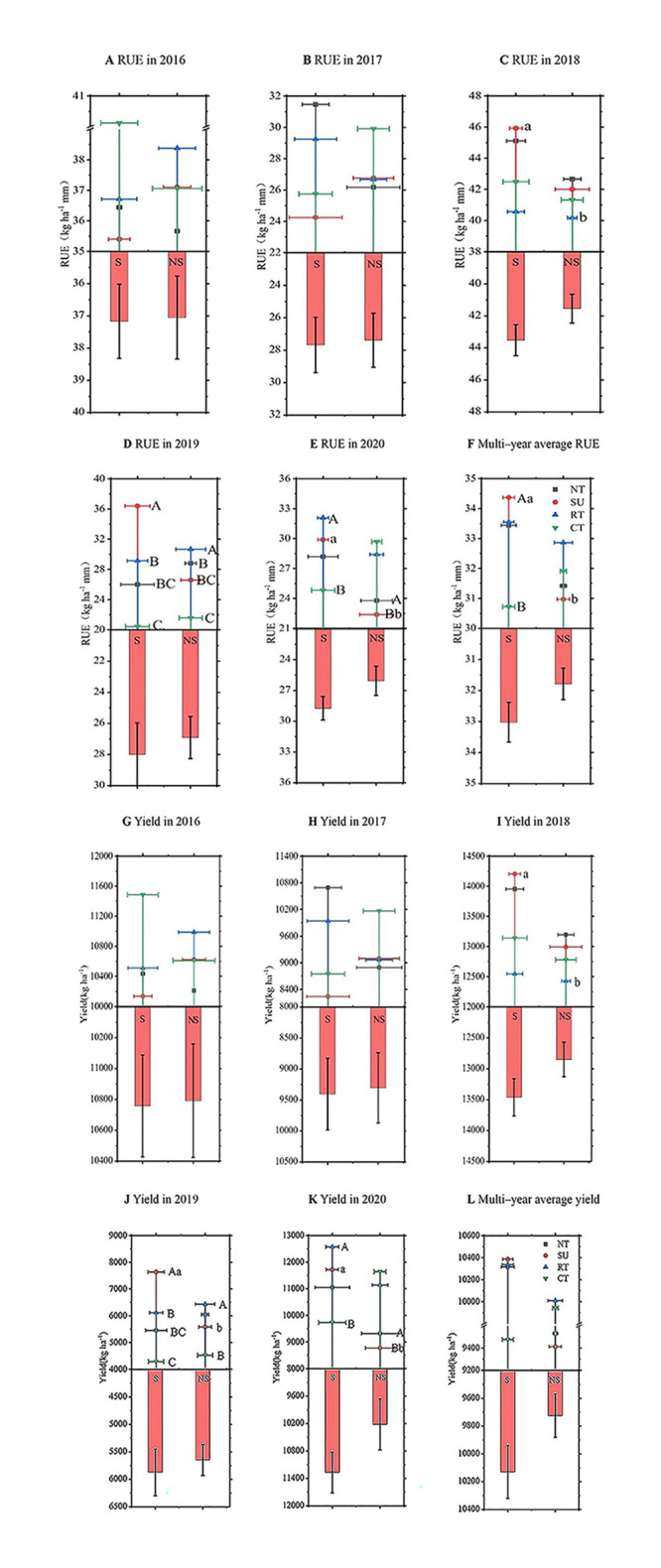
Diagram of RUE and yield affected by tillage cultivation. Note: S, straw return; NS, straw removal. Different capital letters on the error bars indicate significant differences among different tillage cultivations under the same straw treatments (*P* < 0.05, double tailed test), and different lowercase letters indicate the same tillage cultivation with significant differences under different straw treatments (*P* < 0.05, double tailed test). No letters on the error bars indicates no significant differences (*P* > 0.05, double tailed test).

RUE was significantly affected by the different tillage cultivations (Fig 8). In 2016, CTS and SUS were the highest and lowest RUE cultivators with 40.12 and 35.4 kg ha^–1^ mm, respectively. In terms of the long–term series and multi–year average, SUS was the cultivation with the highest RUE with an average increase of 3.65 kg ha^–1^ mm compared to the CTS (control). Over five consecutive years of observation, straw returning improved RUE; compared with straw removal, the annual average RUE of straw returning was 1.23 kg ha^–1^ mm higher. With regards to rainfall pattern with severe stress on crop growth (in 2019), compared to NTS, RTS, and CTS, the RUE of SUS was significantly higher by 10.39, 7.24, and 15.96 kg ha^–1^ mm, respectively. Compared to CTS, the RUE of RTS was higher by 8.72 kg ha^–1^ mm. Compared to NT, SU, and CT, RTS had a significantly higher RUE by 1.85, 4.07, and 9.09 kg ha^–1^ mm, respectively. Compared to CT, NT had a significantly higher RUE by 7.24 kg ha^–1^ mm. In 2020, compared to CTS, the RUE of RTS was significantly higher by 7.26 kg ha^–1^ mm. The RUE of SUS and NT increased by 7.50 and 1.39 kg ha^–1^ mm compared to SU. The annual average RUE of SUS was 3.65 and 3.4 kg ha^–1^ mm higher than that of CTS and SU, respectively.

Crop productivity fluctuations were significantly affected by different tillage cultivations (Fig 8). Generally, crop productivity does not experience large fluctuations and is considered a necessary measure to ensure food security [32,33]. In terms of the immediate effect of different tillage cultivations on yield in 2016, CTS and SUS had the highest and lowest crop yield with 11485.75 and 10135.74 kg ha^–1^. From the analysis of long–term series and multi–year average yield, SUS had the highest yield, increasing with an average of 9.59% compared to CTS (control). Observation of the effect of straw returning on crop yield for five consecutive years showed that the contribution of straw returning to crop productivity was proportional to the increase in positioning test time compared with straw removal. The average annual crop yield of straw returning increased by 4.16%. The analysis of rainfall precipitation patterns with severe stress on crop growth (2019) showed that the yield of SUS was higher than NTS, RTS, and CTS by 39.96%, 24.85%, and 78.12%, respectively. Compared with CTS, the yield of RTS was higher by 42.66%, RT was 42.16% higher than CT, and the crop yield of SUS was higher than SU by 36.84%. In 2020, the crop yield of RTS increased by 29.27% compared with CTS, NT increased by 6.20% compared with SU, and SUS increased by 33.52% compared with SU.

### 3.5 Pearson’s corelation analysis

**Fig 9:**
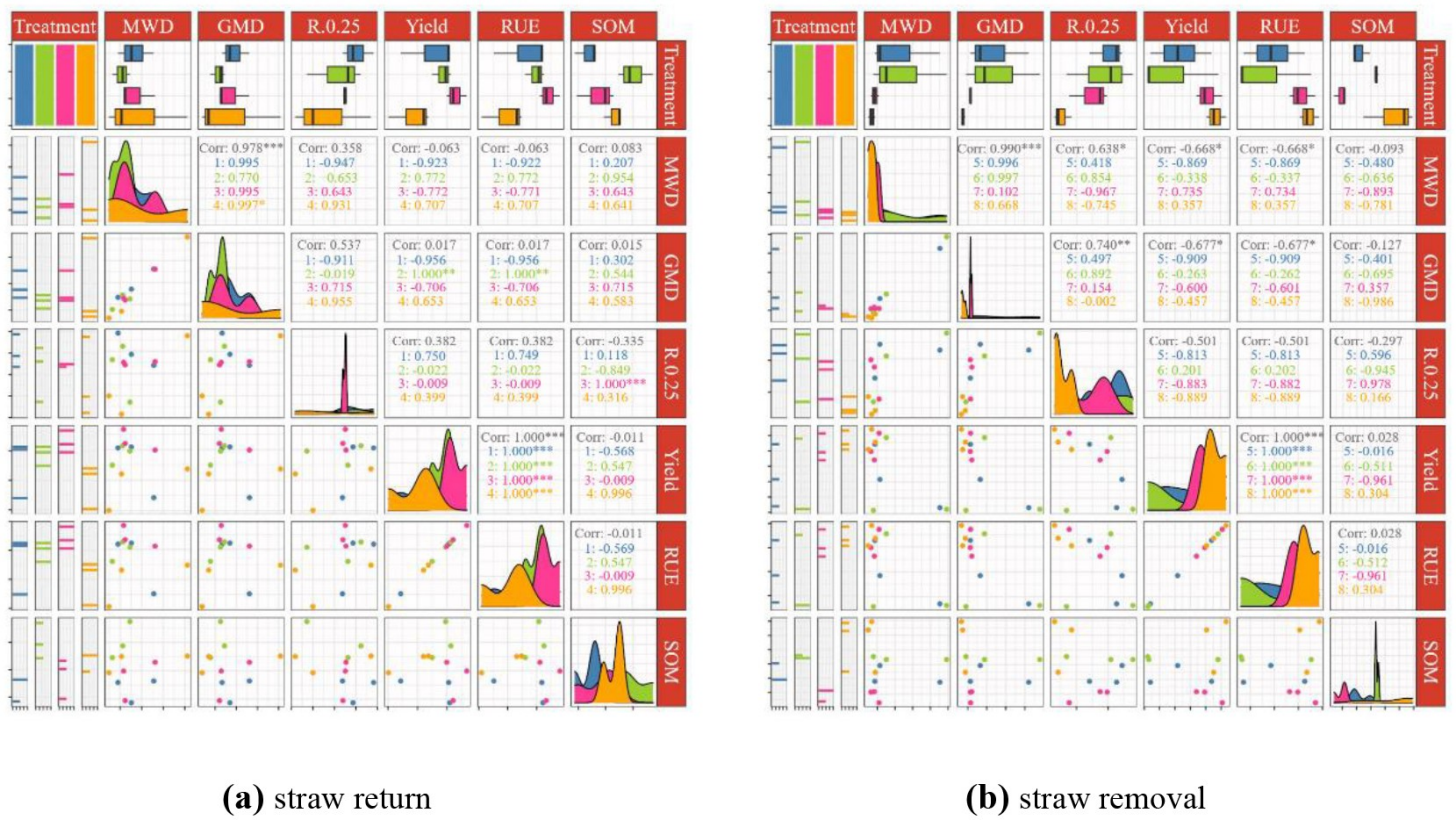
Pearson’s correlation analysis. Note: * *P* < 0.05; ** *P* < 0.01; *** *P* < 0.001; (1) means NTS; (2) means SUS; (3) means RTS; (4) means CTS; (5) means NT; (6) means SU; (7) means RT; (8) means CT.MWD, mean weight diameter. GMD, geometric mean diameter. R_0.25_, >0.25 mm aggregates content. RUE, rainfall utilization efficiency. SOM, soil organic matter. The first column rectangle and its different fillings in the middle indicate different tillage measures and the corresponding eighth column indicator repetition times. Boxplot of second behavior corresponding to tillage and indicator. The lower left is the scatter plot of the corresponding indicator, and the upper right is the correlation coefficient of the two indicators.

Pearson’s correlation analysis [34] of different soil variables influenced by different tillage cultivations is shown in Fig 9. MWD was positively and significantly correlated with GMD, and RUE was significantly positively correlated with yield. Furthermore, GMD was significantly positively correlated with RUE and yield in SUS cultivation. In RTS cultivation, R_0.25_ was positively and significantly correlated with SOM. Fig 9(b) shows the correlation analysis of four tillage cultivations with straw removal. MWD was significantly positively correlated with GMD and R_0.25_ (r ranged from 0.638 to 0.990). MWD was significantly negatively correlated with yield and RUE. GMD was significantly positively correlated with R_0.25_. GMD was significantly negatively correlated with yield and RUE. Yield was significantly positively correlated with RUE.

### 3.6 Interaction of tillage and residue analysis

Soil structure changes effectively when conservation tillage is implemented for four years or more [19]. Therefore, the interaction effect of tillage and straw residue for WSA characterization indexes and SOM was calculated based on analysis of different soil layers, RUE and yield analysis, and on time series analysis based on different years and rainfall patterns. In this study, the interaction effect of tillage and straw residue on SOM in the 20–30 cm soil layer was significant (*P* < 0.05, (Tables 5 and 6)). In addition, the interaction effect of tillage and straw residue in the low rainfall year (2019), which was a stressful year for crop growth, was significant (*P* < 0.05) in hedging the potential risk of low water use efficiency and crop yield reduction due to drought.

**Table 5:** Effects of straw residue and tillage interaction on WSAs and SOM at different soil depths.

indicator	0–10 cm	10–20 cm	20–30 cm	30–40 cm
MWD	*N*	*N*	*N*	*N*
GMD	*N*	*N*	*N*	*N*
R_0.25_	*N*	*N*	*N*	*N*
SOM	*N*	*N*	S*	*N*

Note: N indicates no significant effect (*P* > 0.05, double tailed test); S* indicates significant effect (*P* < 0.05, double tailed test).

**Table 6:** Effect of straw and tillage interaction on RUE and yield in different year corresponding to rainfall precipitation pattern.

indicator	2016	2017	2018	2019	2020
RUE	*N*	*N*	*N*	S*	*N*
Yield	*N*	*N*	*N*	S*	*N*

Note: N indicates no significant effect (*P* > 0.05, double tail); S* indicates significant effect (*P* < 0.05, double tailed test).

## 4. Discussion

### 4.1 Effects of conservation tillage cultivation on improving soil environment

Soil aggregates are well–structured soil microenvironments, and the transition from conventional to conservation tillage improves the soil microenvironment and enhances the stability of aggregates [35]. In this study, SUS and NT significantly enhanced the stability of WSAs in the 20–30 cm and 0–10 cm soil layers, respectively, compared to their respective controls; this is consistent with Zhang et al. [36]. SU was an effective tillage cultivation that broke soil plow layers and, coupled with straw residue, improved the stability of WSAs in deeper soil layers. NT was representative of conservation tillage and it facilitated the enrichment of nutrients in the soil surface layer and had an obvious effect on the stability of this layer [37]. Straw return can increase SOM, loosen the soil, improve its permeability, increase the number of microorganisms, and enhance microbial activity [38]. However, from the perspective of the positioning trial result after five years, straw return does not significantly affect soil structure in conjunction with all conservation tillage effects (Fig 3). The coupling of NT and SU with straw induced limited optimization of soil structure. While the coupling of RT and CT with straw improved soil structure, mainly reason different tillage affected the rate and extent of straw decomposition. NT and SU disturbed the soil less, which was not conducive to straw fragmentation and promoted straw decay. RT rotated and broke the soil, and CT changed the soil sequence in addition to breaking it, which effectively crushed the returned straw and accelerated straw decay.

In the layer–by–layer analysis (Fig 3), it was found that the coupling of NT and SU with straw return mainly increased the content and stability of soil macro–aggregates in the 0–10 cm surface layer, while the coupling of RT, CT, and straw affected deeper soils (0–30 cm), which is consistent with the findings of a long–term localization experiment in northeastern China [39]. From a sustainability point of view, NT and SU in deeper soil accelerate root growth because there is no plow pan left in the soil. RT and CT form a plow pan in the soil, which is not conducive to the formation of a healthy soil structure. In the no–till–deep–pine rotation in the winter wheat–spring maize rotation of the dry plateau of northwest China, the soil in the 0–60 cm soil layer formed a healthy structure [40], which full proved this conclusion.

### 4.2 Effects of conservation tillage cultivation on stabilizing maize yield

Conservation tillage can effectively mitigate the negative effects of stress on crop growth while improving crop yield continuity and stability across different rainfall precipitation patterns (Fig 8). In this study, the coupling effect of straw return and tillage was higher in both the short and long term than yield under tillage alone; furthermore, straw as a natural fertilizer improved resource use efficiency and increased crop yield by enriching the soil [41]. Moreover, in rainfall precipitation year (2019) that stresses on crop growth, SUS was most effective at hedging against drought, stabilizing yield, and avoiding large fluctuations in yield. In semi-arid areas of northwest China, conservation tillage can completely replace conventional tillage resulting in yield increases of 18.00% to 33.26% compared to conventional tillage [42]. In semi-arid Mediterranean agroecosystems in Europe, conservation tillage such as less tillage and less tillage with green manure cover increases yields by reducing soil erosion rates and protecting the soil [43]. No-tillage perform better in yield under rainfed conditions, especially in dry climates mainly because the reduced tillage system retains more soil moisture [44]. Although the global selection of specific measures in conservation tillage needs to be determined by climate and other factors, the global growth of conservation tillage from 45 million hectares in 1999 to 155 million hectares in 2014 [45] indicates the critical role conservation tillage plays in stabilizing crop yields.

### 4.3 Effects of conservation tillage cultivation on hedging against drought

Tillage cultivations can change the hydraulic properties of soil, which may affect its water–holding capacity and hydraulic conductivity [46,47]. Conventional tillage excessively disturbs the soil and exacerbates water stress in agricultural soils [48]. In contrast, SUS and SU, types of conservation tillage, helped to improve the water holding capacity of the soil and stored more water (Figs 4 and 5) [38]. The main difference was that SU increased the water holding capacity of the surface soil (0–20 cm) and SUS increased the water holding capacity of the subsurface soil (20–40 cm). Different tillage cultivations affected the water supply capacity of soil at different depths (Fig 6). NTS had a strong water supply capacity at 0–10 cm, as did RT at 10–20 cm, CTS at 20–30 cm, and SU at 30–40 cm. Although CTS enhanced the water supply capacity of the 20–30 cm soil layer, the upward shift of the soil plow layer increased the root uptake resistance [48]. Under SU, more water can be stored in deeper soils, which promotes root extension to depths [49].

Higher levels of SOM improve the soil–water retention, which may reduce agricultural yield losses due to drought [50]. Proper tillage practice can effectively increase in SOM content, regulating soil C/N, changing soil temperature and moisture, and provide abundant available resources for the growth and reproduction of soil microorganisms [51]. SUS significantly increased SOM content in the 0–20 cm soil layer and contributed the most to SOM enhancement in the 20–40 cm soil layer in this study (Fig 7). This result is consistent with previous research findings wherein SUS tillage increased SOM content by 13.8% compared to CT in arid regions of Northwest China [52]. Frequent soil tillage reduced organic carbon and the structural stability of the soil surface, thus intensifying soil surface loss and nutrient loss in farms [53]. Conservation tillage helps increase soil nutrients and is conducive to increasing the content of alkaline nitrogen, total nitrogen, and organic carbon in soil, thus improving soil quality [54]. Furthermore, several studies found that SUS significantly increased soil urease and sucrase activities. This increase improves soil properties by accumulating crop residues in the surface layer, thus increasing microbial activity and biomass in the soil surface layer, increasing soil carbon, nitrogen, and phosphorus contents, soil moisture, bulk density, agglomeration stability, and soil enzyme activity [55].

The process of implementing different tillage cultivations in order to hedge drought is similar to dominoes. In this study, the direct factors are the direct measures of soil water holding capacity, water supply capacity, and RUE, while the indirect factors are the indirect measures of WSAs and SOM content. The combined forces of different factors eventually affect the results of dominoes.

The correlation analysis showed a significant positive correlation between rainfall utilization efficiency and crop yield, demonstrating that precipitation is a key factor governing yield and that improving precipitation resource use efficiency can increase crop yield [56]. Generally, the larger the MWD and GMD, the better the soil structure, which is more conducive to the improvement of resource utilization efficiency and yield [57]. However, the conclusion of this experiment was contrary, where under the four tillage measures of NT, SU, RT, and CT, the indicators of WSAs were negatively correlated with rainfall utilization efficiency and yield. This may be because changing soil quality is a complex and dynamic process, and we only monitored a small part of it. This is where the special effect mechanism lacks in–depth understanding, and the entire complex process has not yet reached the level of visualization.

## 5. Conclusion

In this study, we investigated the effects of conservation tillage on the quantity and quality of soil aggregates, soil–water holding, water supply capacity, and soil fertility. On this basis, we monitored the annual rainfall pattern from 2016 to 2020 and evaluated the hedging effects of different conservation tillage cultivations on drought and grain yield reduction. We demonstrated that conservation tillage (NTS, SUS, RTS, NT, SU, and RT) can improve the quantity of soil aggregates, enhance the stability of aggregates, improve soil fertility at different soil depths, and hedge drought and grain yield reduction. The results emphasize that SUS increased aggregate content, improved aggregate stability, enhanced soil–water storage, holding, and supply capacity, and improved soil organic matter, and under different rainfall patterns, hedge against drought and grain yield reduction most effectively. SUS, is the more effective conservative tillage in Northern China of great significance in achieving sustainable development of the soil environment, hedging drought, and ensuring food security.

## Supporting information

S1 Data (ZIP)
